# Binding of response-independent task rules

**DOI:** 10.3758/s13423-024-02465-9

**Published:** 2024-02-01

**Authors:** Moritz Schiltenwolf, David Dignath, Eliot Hazeltine

**Affiliations:** 1 Department of Psychology, Eberhard-Karls University of Tübingen, Tübingen, Germany; 2 Department of Psychology, University of Tübingen, Schleichstrasse 4, 72076 Tübingen, Germany; 3 Department of Psychology, University of Iowa, Iowa City, IA USA

**Keywords:** Task switching, Binding, Abstract cognitive control

## Abstract

Binding theories claim that features of an episode are bound to each other and can be retrieved once these features are re-encountered. Binding effects have been shown in task-switching studies with a strong focus on bindings of observable features such as responses. In this study, we aimed to investigate whether task rules, translating stimulus information into motor output can be bound and subsequently retrieved even if they act independently from specific response codes. To address this question, we utilized a task-switching paradigm with varying visual context features. Unlike previous studies, tasks in the present study did not differ in their response options, and sequential response repetitions were eliminated by design. In three experiments, we observed larger task-switch costs on trials repeating the context of the previous trial than on context-change trials. According to binding accounts, this suggests that response-independent task rules adopted in the previous trial became bound to the context feature and were retrieved upon re-encountering the context feature in the current trial. The results of this study generalize previous findings indicating that binding processes can include response-independent control to task-switching situations.

## Introduction

**Fig. 1:**
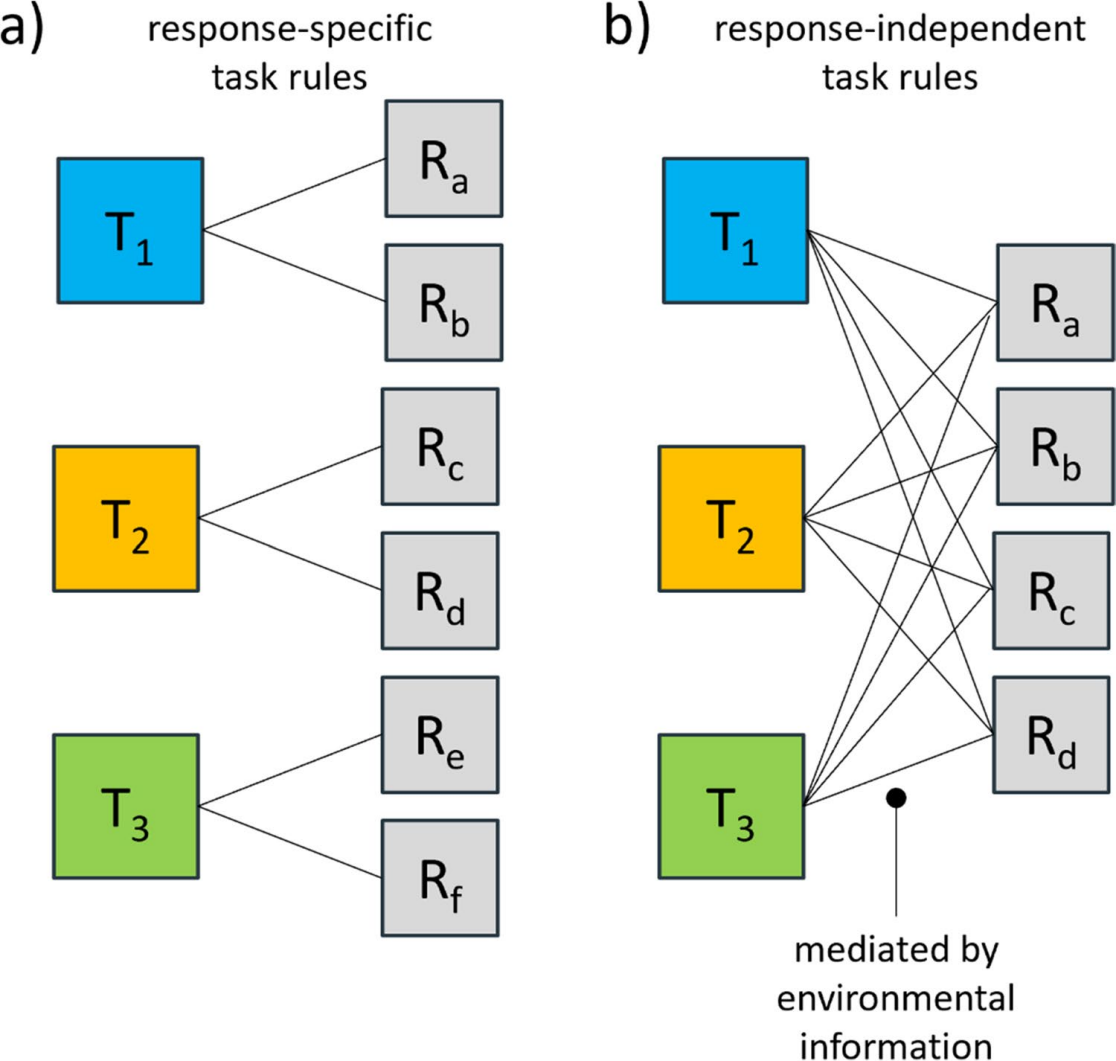
Response-specific and response-independent task rules. (**a**) With response-specific task rules, each task is mapped to specific responses, so the pool of possible responses is constrained by the instructed task. (**b**) With response-independent task rules, all tasks share the same pool of responses. Additional environmental information mediate the mapping of the tasks to the correct responses

Storing current experiences in memory guides future actions. The interplay between integration of sensorimotor information and subsequent retrieval – a core mechanism driving human behavior – is addressed by binding theories (Frings et al., 2020; Hommel et al., 2001). While studies show that features of stimuli and responses can be rapidly integrated into instances of episodic memory and retrieved (e.g., Rothermund et al., 2005), it remains up for debate whether the same binding mechanism also applies to task rules, i.e., the cognitive representation of rules how to translate the stimulus input into correct motor output (e.g., Mayr & Bryck, 2005; Vaidya & Badre, 2022). Such task rules can be conceptualized on a hierarchical scale, where lower level task rules are *response-specific*, meaning that they allow a direct mapping of stimuli to actions, whereas higher level task rules are *response-independent*, since the task itself may not constrain the pool of possible actions, but additional environmental information is required for the stimulus to action translation (Sayalı et al., 2023; see Fig. 1). In contrast to previous work examining response-specific binding effects in task switching (e.g., Kandalowski et al., 2020), the current study focuses on the bindings of task rules that are response-independent. Specifically, we examine whether task rules are bound with visual contexts so that repeating the context allows for subsequent retrieval of these task rules. Critically, we use a paradigm under which such effects cannot reflect the retrieval of responses that are generally linked to a specific task (Oberauer et al., 2013) or previously activated responses (Hommel, 1998).

When people switch between multiple tasks, goal-based behavior requires appropriate task sets to shield and schedule individual tasks (Rogers & Monsell, 1995). While there are different definitions for the term “task set,” many researchers agree that task sets orchestrate the identification of task-relevant stimuli, the selection, and execution of responses (e.g., Schneider & Logan, 2014; Vandierendonck et al., 2010). To study task sets, researchers rely on task-switching paradigms in which participants are required to switch flexibly between different sets of rules (i.e., tasks) to produce the appropriate response (for a review, see Koch et al., 2018b). Examples for such tasks include stimulus classification, arithmetic operations, or spatial operations (Allport et al., 1994; Baddeley et al., 2001; Mayr & Kliegl, 2000).

Switching from one task set to another is a costly process, reflected by worse performance on trials involving a different task from the previous trial than on trials repeating the previous task (switch costs). Traditionally, switch costs are attributed either to a reconfiguration process, during which the new task set needs to be implemented (Rogers & Monsell, 1995; Rubinstein et al., 2001) or to interference between residuals of the previously implemented and the new task-set (Allport et al., 1994). However, binding theories (Frings et al., 2020) offer an alternative interpretation. The basic assumption of these theories is that events are transiently encoded through the features of concurrently perceived stimuli, performed actions, and produced action-effects (Frings et al., 2020; Hommel et al., 2001; see also Kahneman et al., 1992). Hence, when one of the previously bound features is encountered again, all other features that were linked to the repeated feature are retrieved. As a consequence, a match between bound task features with the current task demands (e.g., by activating the correct response) facilitates performance, whereas a feature mismatch requires an updating that impairs performance (e.g., Frings et al., 2015; Rothermund et al., 2005; Foerster et al., 2021; Hommel et al., 2004; Stoet & Hommel, 1999).

Binding perspectives have also inspired recent formalizations of task-sets. For example, Oberauer et al. (2013) postulated that task sets can be described as bindings between stimuli or stimulus categories, corresponding responses, and expected outcomes in working memory. By this account, switch costs are assumed to be the product of interference between currently active bindings and residual activation of outdated bindings, and/or resource intensive memory-updating processes. Another account based on episodic encoding, the Parallel Episodic Processing model (Schmidt et al., 2016), assumes that stimuli, task rules, task decisions, and responses are integrated into memory by an iterative process (Schmidt et al., 2020). This model holds that when both the task cue and the required response repeat, switch costs will be inflated by bindings. This is because the task cue repetition will not only trigger the retrieval of the task rule, but also retrieval of the stimulus and response codes of the previous trial. Therefore, if consecutive trials match on these codes, performance will be facilitated. In contrast, when the task switches, costs can arise from stimulus repetitions because they were bound to different task rules and possibly different responses (Allport & Glenn, 2000; Schmidt & Liefooghe, 2016).

Effects of response bindings on task switching have been tested experimentally by manipulating context features (e.g., Koch et al., 2018a). In task-switching research, context manipulations are often implemented as informative cues for certain task demands. For instance, Crump and Logan (2010) employed the location at which stimuli were presented as context that was correlated with the likelihood of encountering either the same task as the previous trial or a different task. Findings from studies using such informative contexts show that participants learned these contingencies and retrieved context-appropriate control states (see also Chiu & Egner, 2017; Leboe et al., 2008). However, context can also influence behavior in task switching without being directly linked to specific task demands. In contrast to the aforementioned context-correlation design, situations we have in mind are those in which contexts are orthogonal to the specific task demands (i.e., task demands and context are *not* correlated). Since we want to explore such effects in this study, we refer in the following to the term *context* as task features that are not informative about current task demands such as whether a specific task or task switch is to be expected or which response is required. Koch et al., (2018a; see also Kandalowski et al., 2020) used the task-cue modality as context. Although context and responses were uncorrelated, they found that response repetition benefits that are usually observed for task repetitions were restricted to context repetitions. This pattern was also observed for other context features such as visual features (Benini et al., 2022a, 2022b), action effects (Schacherer & Hazeltine, 2022), or language (Benini et al., 2022b). Binding theories explain this by assuming that even task-irrelevant context features are bound with the task-relevant features (Frings & Rothermund, 2017). Trial sequences in which context features change from the previous episode while all other stimulus and response features repeat, yield worse performance than context repetition sequences because features from the old episode unfit for the demands of the current episode might be retrieved or resources must be allocated towards the updating of active bindings (Hommel, 1998; Mocke et al., 2023; Moeller et al., 2016; Rothermund et al., 2005).

Previous research investigating how binding affects task switching has focused on binding and retrieval of specific responses. However, this emphasis on the relation between stimuli and responses hinders a possible generalization of binding mechanisms in task switching. Critically, previous research can only account for binding effects in tasks in which stimuli (categories) map to specific responses (for an in detail discourse, see Hazeltine & Schumacher, 2016). This is important because several studies indicate that task sets incorporate task rules. Our use of the term task rule derives from work of Mayr and Bryck (2005), and refers to the translation of stimulus input into motor output on a more abstract level than simple stimulus-response mappings. For instance, Mayr and Bryck introduced a task-switching paradigm in which switch costs were observed although task switch/repetition sequences used the exact same stimuli and responses, suggesting that switch costs arise due to task rules that provide an appropriate link between stimuli and responses (see also Waszak et al., 2003, Exp. 5). Analysis of neurophysiological and behavioral data of participants performing such a task-switching paradigm suggests that only the strength of EEG-correlates representing bindings between stimuli, responses, and task rules predict behavioral binding effects, not those including only stimuli and responses (Kikumoto & Mayr, 2020). Finally, Haynes et al. (2007) used a voluntary task-switching paradigm in which the two arithmetic tasks shared the same stimulus and response options. The researchers were able to predict the to-be-performed task during the preparation period from decoded brain activity measured with fMRI. Since these results cannot stem from task-specific stimulus or response-code activation, it suggests that task rules can be differentiated on a neural level.

These studies indicate that task sets include more than specific stimulus to response mappings but also comprise the task rules that control correct stimulus to response translation. In other words, to perform a task it is not sufficient to identify the relevant stimuli and responses; it is also necessary to have the correct task rules active, especially when multiple tasks overlap in the pool of stimuli and responses relevant to them. However, studies investigating binding effects on task switching have focused on the retrieval of responses (e.g., Benini et al., 2022a; Kandalowski et al., 2020), and thus it remains unclear whether response-independent task rules can be part of bindings (Egner, 2023). In this regard, it is notable that in the related field of conflict adaptation, studies have shown that cognitive states that control attentional weights independently from specific stimulus or response codes can be bound to context features and retrieved upon context repetitions (Dignath et al., 2019; Dignath & Kiesel, 2021; Grant et al., 2021; Spapé & Hommel, 2008; for theoretical perspectives, see Egner, 2014, 2017), but whether this applies to task-rule binding is unexplored.

**Fig. 2:**
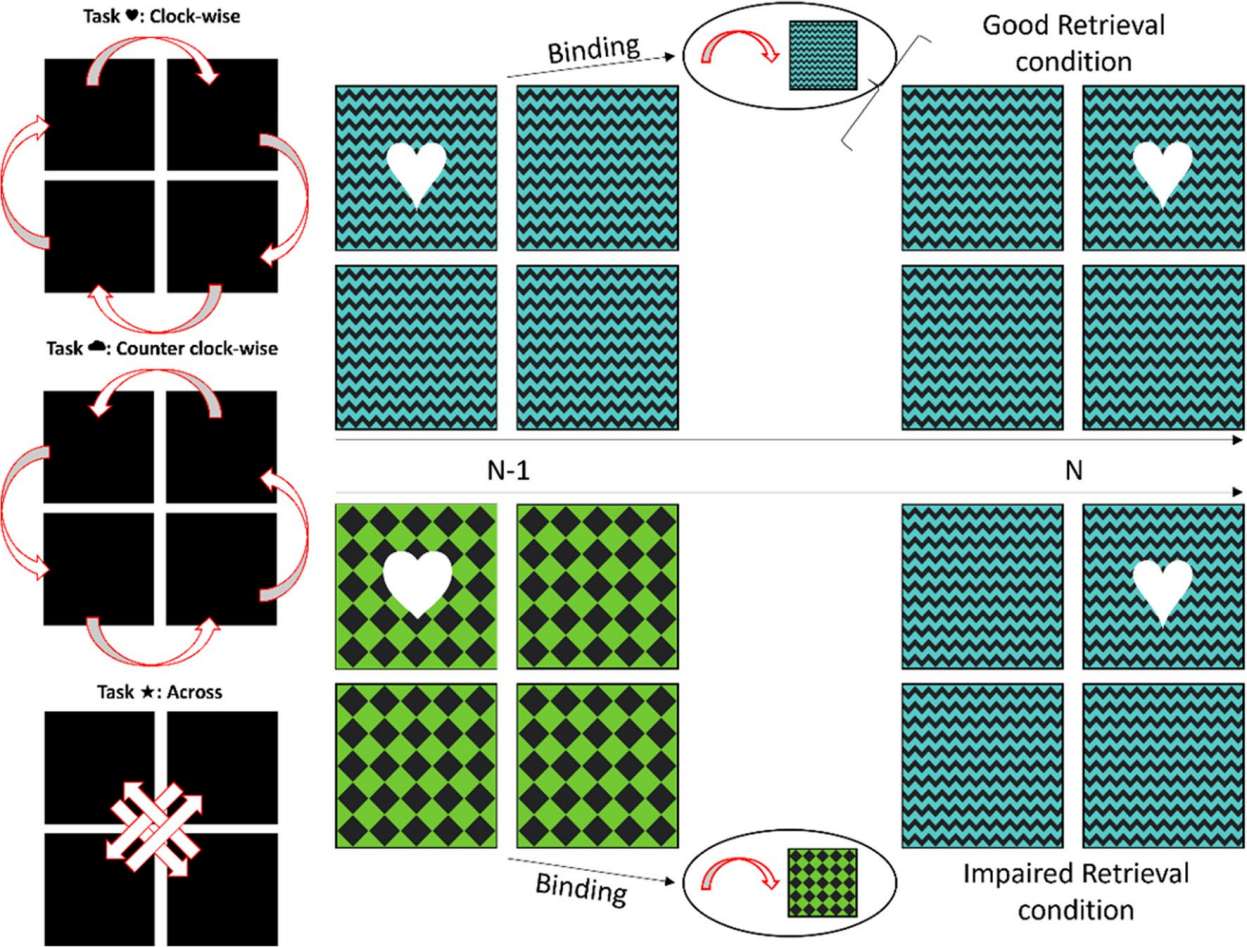
Task rules and context transitions. On the left side of the figure, the goal boxes for each of the three used tasks (clockwise, counterclockwise, and across) are visualized in dependency of the starting box. On the right side, an example context transition across sequential trials is shown visualizing the expected binding between the task rules and the visual context feature

Here, we address this question by testing whether response-independent task rules that guide the translation of stimulus input into response output can become bound and retrieved. We used a task-switching paradigm similar to that of Mayr and Bryck (2005; see also Kikumoto & Mayr, 2020; Rangel et al., 2023) in which participants performed one of three spatial operation tasks (Fig. 2). A strength of this paradigm is that it controls for the impact of stimulus and response bindings on switch costs (Schmidt & Liefooghe, 2016), since the tasks cannot be distinguished by specific response mappings or sets and response repetitions across trials can be avoided in an intuitive way. Consequently, task-switch costs should reflect costs of switching task rules that translate the stimulus setup into an appropriate response.

To assess whether such task rules can be bound and retrieved, we presented visual context features (colorized background patterns) that either repeated or changed across consecutive trials. According to binding theories, response-independent task rules become bound to the context feature and are retrieved when the same context feature reappears (Frings et al., 2020). If the context from the preceding trial repeats, this should result in pronounced switch costs since retrieving the correct task rules should facilitate performance on task repetitions, but on task switches, performance should be impaired as mismatching task rules are retrieved. Therefore, we predicted larger switch costs on trials where the context repeats from the previous trial compared to trials where the context changes (see Fig. 2). To test this prediction, we conducted three structurally identical experiments. In Experiment 1, we used a trial order resulting in 50% task and context repetitions, while in Experiment 2 the chance for each task and context combination was independent from the previous trial (which equals 33% task and context repetitions). In Experiment 3, we controlled for stimulus-to-stimulus bindings between the context and the task cues by mapping two task cues to each task and ensuring that task cues never repeat across trials.

## Experiment 1

### Methods

The hypothesis, procedure, outlier criteria, methods, and planned analysis were preregistered on the Open Science Framework (OSF; https://osf.io/rb73g). Raw data, scripts for the experiments, and analysis are available on the OSF.

#### Participants

We analyzed a sample of 45 participants (11 female, 31 male; mean age: 28 years; three participants provided no demographic information). All participants were recruited on Prolific (Palan & Schitter, 2018) and were in the age range of 18–40 years, had German as their first language, and had no issues seeing colors. A pilot study indicated an effect size for task binding of d_z_ = 0.968, which would require a sample size of 14 participants to achieve a test power of 95% with a .5 alpha criterion. Since the pilot study used a different task and context manipulations, we decided to increase the sample size. No participant was excluded from the analysis.

#### Task and stimuli

The experiment was coded for a browser environment using the JavaScript-based library jsPsych (de Leeuw, 2015). During the experiment, four black boxes were continuously displayed in a 2 x 2 grid. One of the boxes was the starting box for the current trial, and the participants were instructed to identify the correct goal box depending on the indicated task rule. The goal box of the current trial always was the starting box for the next trial.

Each trial followed this structure (display duration in parentheses): Fixation cross without context (500 ms), fixation cross and context onset (500 ms), blank (35 ms), task cue (1,500 ms or until a response was given). At the beginning of each trial, a fixation cross was presented in the starting box. Upon context onset, the background of all boxes was filled with one of the three colorized context patterns (green chess board, yellow serpentines, blue zigzags), which lasted until the end of the trial. During the blank neither the fixation cross nor the task cue were visible. The task cue was presented superimposed and centrally in the same box as the fixation cross indicating which of the three tasks the participant had to perform in the current trial. The task rules were *clockwise* (correct response is the next box in clockwise direction; indicated by “♥”), *counter-clockwise* (next box in counter-clockwise direction; indicated by “☁”), and *across* (box on the diagonal opposite side; indicated by “★”). Depending on the task, the participants had to decide which of the boxes would be the correct goal box and provide the response via key press (top left box: Key “R” with left middle finger; bottom left box: Key “F” with left index finger; top right box: Key “T” with right middle finger; bottom right box; Key “G” right index finger). Giving a response ended the current trial. Giving no or an incorrect response within the stimulus duration was registered as error; feedback was presented for 1,500 ms (the screen turned red and “WRONG BOX!” on normal trials or “PAY ATTENTION TO COLOR AND SHAPE!” on catch trials was presented in German centrally on the screen). Since the starting box of each trial was goal box of the previous trial, sequential trials never required the same response. To ensure that participants attend to contexts, we added catch trials on 10% of the trials. A catch trial was indicated by either the context pattern (dots) or color (pink), and the task was not to respond to the task cue but to press the space bar with the thumb.

#### Procedure

The experiment was conducted online on the private devices of the participants. A minimum browser resolution of 1,280 x 700 px was required to start the experiment. After providing informed consent, the participants received instructions and performed a training block. If participants failed to provide at least six correct responses in the first ten trials of the training block, instructions were presented again. If they failed this attention check a second time, the experiment was terminated. After finishing 43 training trials, participants worked on ten experimental blocks each containing 64 trials. Trial order was determined by an algorithm so that N-2→N-1 task transitions, N-1→N task transitions, N-2→N-1 context transitions, N-1→N context transitions were orthogonally balanced, i.e., each combination of these factors appeared equally often per block. Each participant was paid £4.50 after finishing the experiment.

### Results

Before analysis, we applied the preregistered trial outlier criteria and excluded all catch trials, trials following catch trials, the first trial of each block, trials involving backward inhibition task sequences (A→B→A tasks sequences, see, e.g., Koch et al., 2010), and trials following error trials from analysis. For response time (RT) analysis, we also excluded error trials and trials deviating more than 3 SD from the individual factorial cell mean. In total we excluded 31.5% of the trials from analysis.

**Fig. 3:**
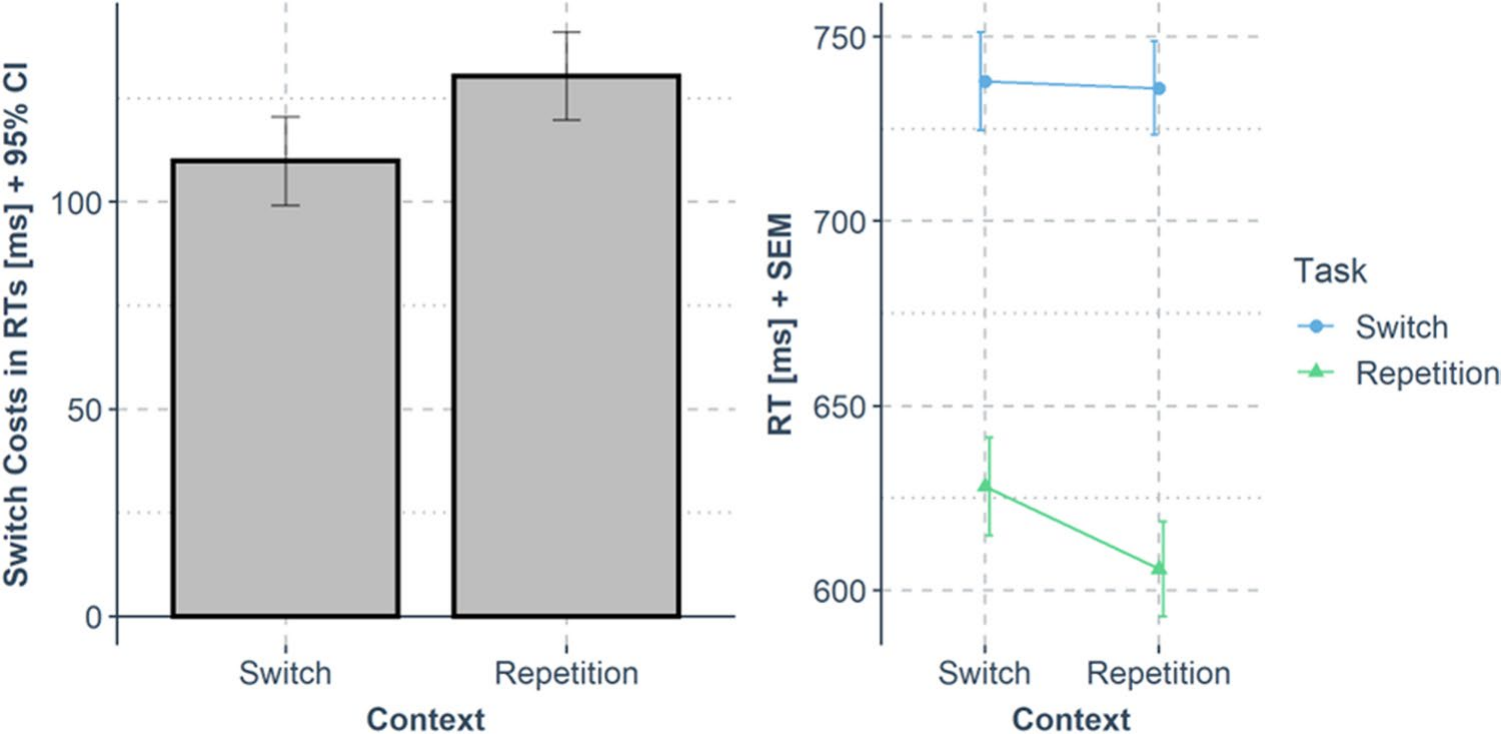
Results from Experiment 1. The left panel shows switch costs in response times (RTs) (*y*-axis; calculated as mean RT_task switch_ – mean RT_task repetition_; error bars indicate the 95% confidence interval for the paired differences) dependent on the context transition (*x*-axis). The right panel shows the same data in mean RTs (*y*-axis; error bars indicate the standard error of the mean for each condition) dependent on the context transition (*x*-axis) and task transition (color)

A repeated-measures ANOVA with the factors task transition [task repetition vs. switch] and context transition [context repetition vs. change] was conducted for RTs and error rates. The RT results are visualized in Fig. 3 ﻿and Table 1.

**Table 1:** Mean response times (RTs) and error rates for each trial condition in Experiments 1, 2, and 3 and the resulting switch costs

	Experiment 1		Experiment 2		Experiment 3	
	RT in ms	Errors in%	RT in ms	Errors in%	RT in ms	Errors in%
*Context repetition*						
Task repetition	606 [± 12.8]	3.6 [± 0.5]	702 [± 10.5]	4.6 [± 0.4]	745 [± 10.6]	4.2 [± 0.4]
Task change	736 [± 12.8]	7.4 [± 0.9]	773 [± 11.0]	4.7 [± 0.3]	874 [± 10.7]	6.4 [± 0.5]
Switch Costs	130 [± 6.7]	3.7 [± 0.7]	71 [± 5.4]	0.1 [± 0.4]	129 [± 8.5]	2.2 [± 0.5]
*Context change*						
Task repetition	628 [± 13.3]	3.5 [±0.4]	716 [± 10.1]	4.2 [± 0.2]	766 [± 9.9]	4.5 [± 0.3]
Task change	737 [± 13.3]	7.2 [±0.9]	774 [± 11.3]	4.9 [± 0.3]	881 [± 10.7]	5.9 [± 0.4]
Switch Costs	110 [± 6.6]	3.7 [± 7.6]	58 [± 5.0]	0.7 [± 0.3]	115 [± 6.3]	1.4 [± 0.4]

Switch costs were calculated as RT/error_task change_ – RT/error_task repetition_. Standard errors are given in brackets

**Response times (RTs).** We observed two main effects: A main effect of task transition, *F*(1, 44) = 382.43, *p* < .001, *η*p2 = .897, because RTs in task repeat trials were shorter (*M* = 617 ms) than RTs in task-switch trials (*M* = 737 ms), and a main effect of context transition, *F*(1, 44) = 25.84, *p* < .001, *η*p2 = .370, because RTs in trials that repeated the context of the previous trial were shorter (*M* = 671 ms) than in trials with a different context (*M* = 683 ms). Most importantly, a significant two-way interaction between the factors task and context transition was observed, *F*(1, 44) = 16.25, *p* < .001, *η*p2 = .270, because task-switch costs were higher in trials that repeated the context of the previous trial (Δ = 131 ms) than in trials with a different context (Δ = 109 ms).

**Errors.** We observed a main effect of task repetition, *F*(1, 44) = 26.71, *p* < .001, *η*p2 = .378, because error rates in trials that repeated the task of the previous trial were lower (*M* = 4%) than in trials with a different task (*M* = 7%). No other effect was significant (*p* ≥ .540).

## Experiment 2

An important difference between task-switching paradigms using only two tasks and those using three tasks (as in this study) is that the conditional probabilities for the occurrence of not performed tasks in task-switch trials differ. If only two tasks are possible, a task switch necessarily means a switch to the previously not performed task, whereas if three tasks are possible on a task switch there is a 50% chance for each of the previously not performed tasks to occur. Thus, balancing task repetitions and switches (as in Experiment 1) means that the probability that the same task occurs as in the previous trial was 50%, but the probability for each of the remaining tasks was only 25%. This imbalance may have given participants an incentive to prepare the previously performed task, since out of the three it was the most likely task to occur. In Experiment 2, we aimed to replicate the findings of Experiment 1, but, instead of equally balancing the probability for task and context transitions, we balanced the trial order so that each of the three tasks and contexts could appear with the same probability. In this way, the chance for each task and context to occur was independent from the previous trial and therefore, there was no incentive to prepare the previous tasks or contexts.

### Methods

The hypothesis, procedure, outlier criteria, methods, and planned analysis were preregistered on the OSF (https://osf.io/ktxrm). Raw data, scripts for the experiments, and analysis are available on the OSF.

#### Participants

We analyzed a sample of 104 participants (45 female, 55 male, four diverse; mean age: 27 years). A power analysis indicated a sample size of *N* = 103 to achieve a test power of 90% with a .5 alpha criterion to observe an effect size of d_z_ 0 .291. This effect size was estimated based on N-2→N binding and retrieval effects (see Discussion) observed in a pilot study. One participant was collected additionally due to a technical error. The recruitment criteria were identical to Experiment 1, but participants who took part in Experiment 1 were excluded. Two participants were excluded from analysis due to an error rate higher than 30%. Both participants were replaced.

#### Task, stimuli, and procedure

The experiment was structurally identical to Experiment 1, but the probability for task and/or context repetition across trials was reduced to 33% (in Experiment 1: 50%).

### Results

**Fig. 4:**
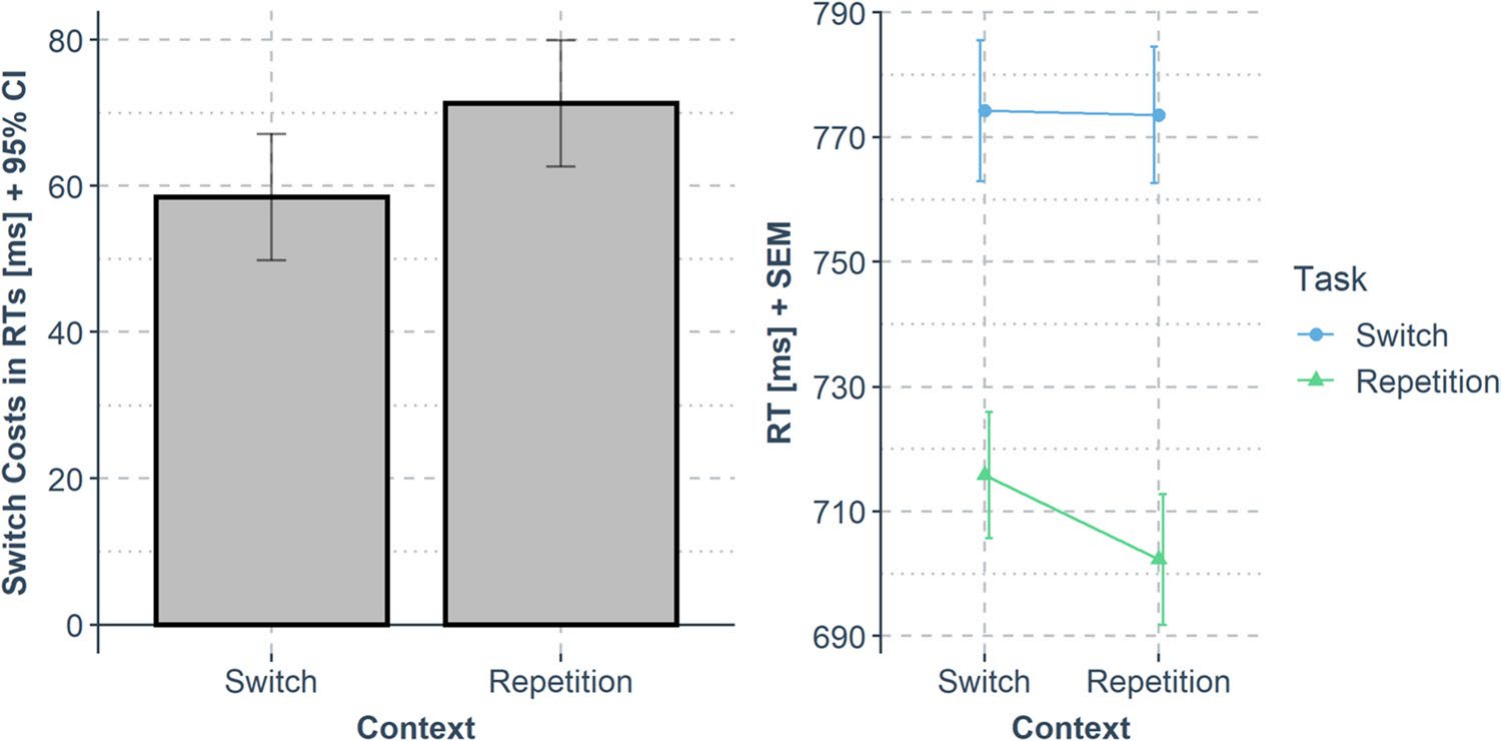
Results of Experiment 2*.* The left panel shows switch costs in response times (RTs) (*y*-axis; calculated as mean RT_task switch_ – mean RT_task repetition_; error bars indicate the 95% confidence interval for the paired differences) dependent on the context transition (*x*-axis). The right panel shows the same data in mean RTs (*y*-axis; error bars indicate the standard error of the mean for each condition) dependent on context transition (*x*-axis) and task transition (color)

The same analysis plan as for Experiment 1 was conducted for Experiment 2 (45.5% of the trials excluded from analysis due to the preregistered exclusion criteria). The RT results are shown in Fig. 4 ﻿and Table 1.

#### RTs

We observed two main effects: A main effect of task transition, *F*(1, 103) = 188.43, *p* < .001, *η*p2 = .647, because RTs in task repeat trials were shorter (*M* = 709 ms) than in task-switch trials (*M* = 774 ms), and a main effect of context transition, *F*(1,103) = 10.03, *p* = .002, *η*p2 = .089, because RTs in trials that repeated the context of the previous trial were shorter (*M* = 738 ms) than in trials with a different context (*M* = 745 ms). Finally, a significant two-way interaction was observed, *F*(1, 103) = 8.61, *p* < .001, *η*p2 = .077, because task-switch costs were larger in trials that repeated the context of the previous trial (Δ = 72 ms) than in trials with a different context (Δ = 58 ms).

#### Errors

The same analysis on error rates resulted in no significant effect (*p* ≥ .138).

## Experiment 3

Experiments 1 and 2 used one task cue for each task, so task repetitions were also task cue repetitions. Empirical studies have shown that a performance benefit in cue repetition trials exists beyond switch costs (Forstmann et al., 2007; Logan & Bundesen, 2003; Mayr & Kliegl, 2003) and both processes are dissociable on a neurophysiological level (Jost et al., 2008). Further, it has been suggested that retrieving visual stimulus features independently from response features can improve performance if the retrieved stimulus features match the currently perceived stimulus features and impair performance if there is a feature mismatch (Giesen & Rothermund, 2014). Regarding Experiments 1 and 2, both described mechanisms could provide an alternative explanation to bindings between response-independent task rules and the context: Either participants may have been able to encode task cues faster if the context repeated, or stimulus-to-stimulus bindings between the task cue and the context supported the processing of the task cue. To address these alternative explanations, we conducted a third experiment in which two task cues were mapped to each task. Trial order was adjusted so that task cues never repeated across trials. Because the task cue always changed, binding effects cannot be the result of visual encoding benefits, or bindings between the task cue and the context.

### Methods

The hypothesis, procedure, outlier criteria, methods, and planned analysis were preregistered on the OSF (https://osf.io/nryw8). Raw data, scripts for the experiments, and analysis are available on the OSF.

#### Participants

Following the same sample size reasoning as in Experiment 2, we collected a sample of 103 participants (47 female, 52 male, four diverse; mean age: 28 years). The recruitment criteria were identical to the previous experiments. One participant was replaced due to an error rate higher than 30%.

#### Task, stimuli, and procedure

The experiment was structurally identical to the previous experiments. The main difference was that we used a 2:1 task cue to task mapping and adjusting trial order so that task cues never repeated across trials. Following the largest reported effect size for switch costs with 2:1 mappings in the work of Schneider and Logan (2011), we used semi-explicit task cues: “I” or “M” for *clockwise* (in German “IM Uhrzeigersinn”), “G” or “E” for *counter-clockwise* (“GEgen Uhzeigersinn”), and “K” or “R” for *across* (“KReuzweise”). The probability of context repetitions across trials was 33%, while the probability for task repetitions across trials was 50%.

### Results

**Fig. 5:**
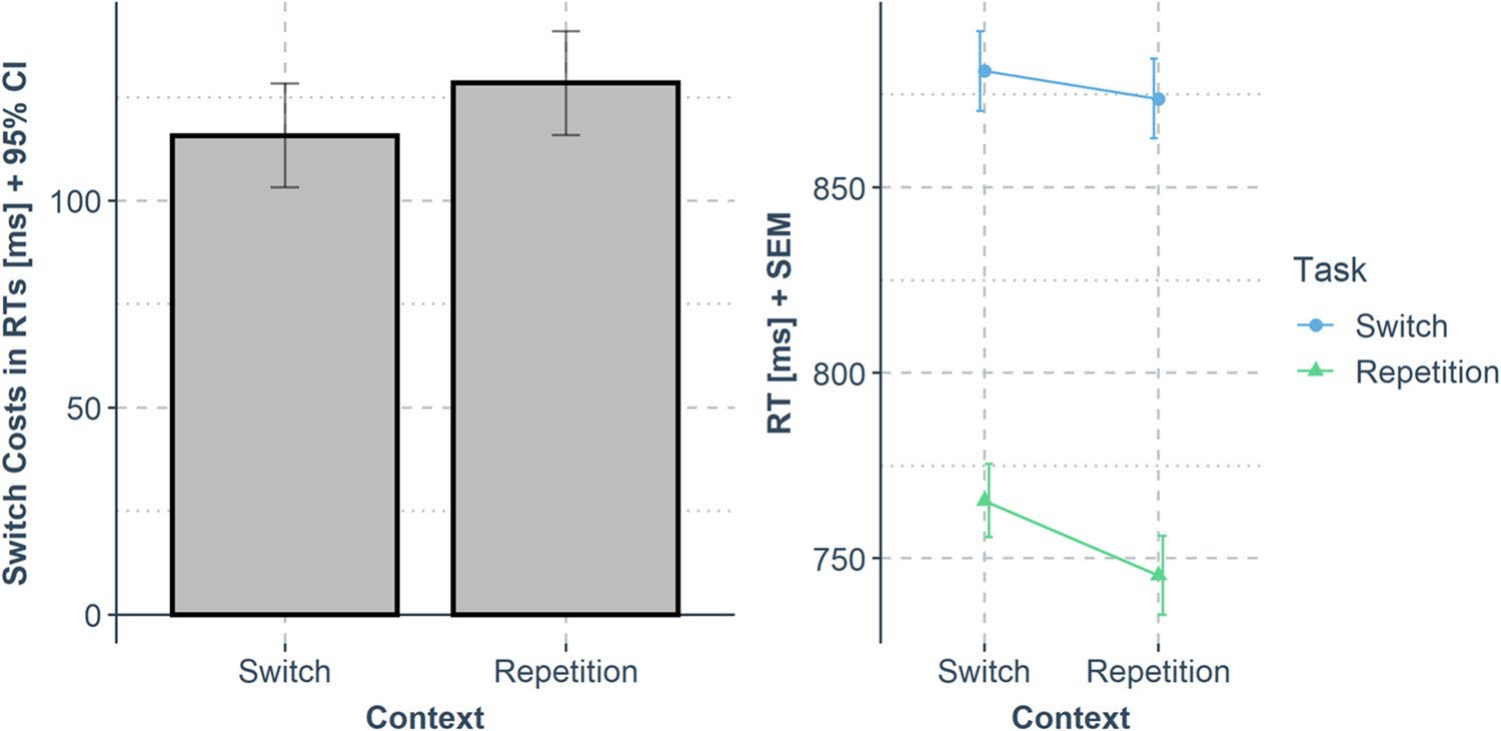
Results of Experiment 3. The left panel shows switch costs in response times (RTs) (*y*-axis; calculated as mean RT_task switch_ – mean RT_task repetition_; error bars indicate the 95% confidence interval for the paired differences) in dependency of the context transition (*x*-axis). The right panel shows the same data for mean RTs (*y*-axis; error bars indicate the standard error of the mean for each condition) as a function of context transition (*x*-axis) and task transition (color)

The analysis plan remained identical as in the previous experiments (37.1% of the trials excluded from analysis due to the preregistered exclusion criteria). The RT results are visualized in Fig. 5 and Table 1.

#### RTs

We observed two main effects: A main effect of task transition, *F*(1, 102) = 325.61, *p* < .001, *η*p2 = .761, because RTs in task repeat trials were shorter (*M* = 756 ms) than in task-switch trials (*M* = 878 ms), and a main effect of context transition, *F*(1,102) = 19.87, *p* < .001, *η*p2 = .163, because RTs in trials that repeated the context of the previous trial were shorter (*M* = 810 ms) than in trials with a different context (*M* = 823 ms). Finally, a significant two-way interaction was observed, *F*(1, 102) = 4.02, *p* = .048, *η*p2 = .038, because task-switch costs were larger in trials that repeated the context of the previous trial (Δ = 129 ms) than in trials with a different context (Δ = 115 ms).

#### Errors

The same analysis on error rates resulted in a significant main effect of task repetition, *F*(1, 102) = 22.37, *p* < .001, *η*p2 = .180, because error rates in task repeat trials were lower (*M* = 4.4%) than in task-switch trials (*M* = 6.2%). No main effect of context transition was observed (*p* > .74). Descriptively, the two-way interaction in error rates showed the same data pattern as in RTs, but the effect was not statistically significant, *F*(1, 102) = 3.07, *p* = .083, *η*p2 = .029.

## Discussion

The current study tested whether task rules that guide the translation of stimulus input into motor output can be bound and retrieved. Importantly, these task rules act independently from specific response codes, i.e., benefits for task repetitions are observable even without response repetitions. In three experiments, we used a task-switching paradigm with three spatial operation tasks and combined it with a visual context feature. According to binding theories adopted task rules and context features should be bound and repeating the context feature in the next trial should facilitate the retrieval of these task rules (Frings et al., 2020). Consequently, on context repetitions, performance on task repetitions should be improved compared to task switches, i.e., the costs to switch tasks should be larger. In line with these predictions, we found that in all three experiments there was an increase in switch costs on context repetition trials. Critically, due to the design, tasks could not be distinguished by their response options. Going beyond previous research (e.g., Koch et al., 2018a; Schuch & Keppler, 2022), the observed increase of switch costs in context repetition trials therefore cannot be attributed to response retrieval. Instead, we suggest that context repetitions facilitated the retrieval of response-independent task rules.

In Experiments 1 and 2, a single task cue was mapped to each task. Consequently, task repetitions were also task cue repetitions. Encoding benefits that result from the context being repeated together with the task cue (Jost et al., 2013), or bindings between the context and the task cue (Giesen & Rothermund, 2014) provide alternative explanations to the observed binding effect. To address these alternative accounts, we mapped two task cues to each task and avoided task cue repetitions by design in Experiment 3. We successfully replicated the binding effect of the first two experiments, which strengthens the conjecture that response-independent task rules can become bound to context features. Although descriptively the observed binding effect was smaller in Experiment 3 (Δ = 14 ms) than in the previous experiments (Exp. 1 Δ = 20 ms; Exp. 2 Δ = 23 ms), no significant difference in the binding effect between the experiments was observed when combining all data in a single ANOVA with experiment as a between-subjects factor.

Showing that task rules can be bound and retrieved generalizes the notion of binding mechanisms to more abstract, non-perceivable aspects of task sets going beyond previous research investigating effects of stimulus and response bindings in task switching (e.g., Koch et al., 2018a; Schuch & Keppler, 2022). Further, this provides an ecologically more valid perspective on the interplay of task switching and binding processes: Repeating the same task only rarely entails an exact repetition of the previous action. Rather, novel actions to novel stimuli must be performed in service of reaching an unchanged (i.e., repeated) task rule as the appropriate action must be selected under consideration of other environmental information (after performing the task “picking flowers,” a task repetition necessarily requires a translation of the environmental information into new actions, since exact action repetitions will only lead you to the exact same spot where no more flowers are left).

Further, the observed effects of bindings between task rules and context features are in line with findings of studies using similar approaches to investigate context effects on other response-independent cognitive states such as attentional weights. For example, in response-conflict paradigms (such as the Flanker task), the *Congruency Sequence Effect,* a behavioral effect supposedly reflecting control adaptation (Egner, 2017), is significantly larger if task-context features repeat across trials than if they change. This effect is attributed to bindings between context features and cognitive parameters controlling the attentional weights that are allocated towards distractor and target information (Dignath et al., 2019; Dignath & Kiesel, 2021; Dignath et al., 2021; Grant et al., 2021; Jiang et al., 2015; Spapé & Hommel, 2008).

It remains an open question to what extent bindings such as those operationalized in this study relate to other forms of contextualized control, in which typically context is instructive of task demands and these contingencies can be learned over time (Bugg et al., 2011; Crump et al., 2006; Crump & Logan, 2010; for reviews see Bugg, 2017; Bugg & Crump, 2012). Oberauer et al. (2013) have developed a computational models of task control with two learning systems: one for fast-changing bindings between task features that hold active in working memory, and one for slow-changing associations in long-term memory. Here the more recent experiences can have a strong influence on behavior via bindings, but they transfer slowly to long-term memory. Following a similar idea, Giesen et al. (2020) found that the episodic retrieval of stimulus-response bindings provide access to the most recent occurrence of the current situation. Such approaches could be employed to test to which extend bindings can account for contingency learning between contexts and task demands (see above) or one-shot learning of context to control associations (Brosowsky & Crump, 2018; Whitehead et al., 2020).

Two limitations of the present study should be noted. First, in all three studies, task-switch sequences appeared unaffected by context transitions, i.e., performance was not impaired on context repetitions compared to context changes, as predicted by binding theories (Frings et al., 2020). Two explanations for this pattern seem plausible. One possibility is that we observed an effect of context transitions beyond the presumed effect of context to task rule bindings. While task rule binding predicts that task switches should be more difficult on context repetitions than on context changes, it's conceivable that the repetition of the context facilitates performance. One possible explanation for the observed benefits of context repetition is that it might provide improved encoding conditions. Alternatively, participants could spend time scrutinizing the constellation of context features to detect catch trials, and this process is likely to be faster if the context repeats than if it changes. Such an overlap of two effects could descriptively offset binding effects in task switch sequences but amplify them in task repetition sequences. On the other hand, it's also possible that we observed an interaction effect between the factors of task transition and context transition, and this interaction was primarily driven by the interplay of task repetitions and context transitions. This perspective challenges the assumption that, on task-switch trials with context repetitions, the task rules from the previous trial are retrieved. Alternative explanation could be that a changing context may disrupt actively maintained task rules (for such a perspective in conflict adaptation, see Kreutzfeldt et al., 2016). With the current dataset, we cannot definitively exclude either possibility, but studies utilizing electrophysiological measures to investigate task rule bindings have begun to explore the retrieval process (Rangel et al., 2023). Possibly, similar approaches could help to determine whether the effects of context to task rule bindings should be described as a result of retrieval processes or alternative mechanisms, such as disruption.

A second limitation could be that previous research indicates that the processing of contextual novelty shares neural networks with task updating processes (Barcelo et al., 2006) and error processing (Wessel et al., 2012). Assuming that participants are biased to expect more context repetitions, contextual changes might elicit surprise and thereby impair performance, which is potentially mostly reflected in the faster task repetition trials. However, this explanation appears unlikely because binding effects were also observed in Experiment 2 in which context repetitions were less likely than context changes. Thus, one would have to make the additional assumption that the repetition bias in expectations was independent from the actual proportion of context transitions.

To sum up, in three experiments, we observed increased switch costs in trial sequences repeating a visual context feature compared to context changes. Since the design of the paradigm controls for response retrieval, we suggest that the observed effects result from bindings between the visual context and response-independent task rules that guide the translation of stimulus input into response output. These findings add to the growing body of literature on the interplay of task switching and binding processes by demonstrating that task rules can be bound and retrieved independently of specific response codes.
